# Complex effects of macrolide venturicidins on bacterial F-ATPases likely contribute to their action as antibiotic adjuvants

**DOI:** 10.1038/s41598-021-93098-8

**Published:** 2021-07-01

**Authors:** Yakov M. Milgrom, Thomas M. Duncan

**Affiliations:** Department of Biochemistry & Molecular Biology, SUNY Upstate Medical University, 750 E Adams St, Syracuse, NY 13210 USA

**Keywords:** Biochemistry, Microbiology

## Abstract

Bacterial energy metabolism is now recognized as a critical factor for the efficacy of antibiotics. The F-type ATPase/ATP synthase (F_O_F_1_) is a central player in cellular bioenergetics of bacteria and eukaryotes, and its potential as a selective antibiotic target has been confirmed by the success of bedaquiline in combatting multidrug-resistant tuberculosis. Venturicidin macrolides were initially identified for their antifungal properties and were found to specifically inhibit F_O_F_1_ of eukaryotes and bacteria. Venturicidins alone are not effective antibacterials but recently were found to have adjuvant activity, potentiating the efficacy of aminoglycoside antibiotics against several species of resistant bacteria. Here we discovered more complex effects of venturicidins on the ATPase activity of F_O_F_1_ in bacterial membranes from *Escherichia coli* and *Pseudomonas aeruginosa*. Our major finding is that higher concentrations of venturicidin induce time– and ATP–dependent decoupling of F_1_-ATPase activity from the venturicidin-inhibited, proton-transporting F_O_ complex. This dysregulated ATPase activity is likely to be a key factor in the depletion of cellular ATP induced by venturicidins in prior studies with *P. aeruginosa* and *Staphylococcus aureus*. Further studies of how this functional decoupling occurs could guide development of new antibiotics and/or adjuvants that target the F-type ATPase/ATP synthase.

## Introduction

The F-type ATPase/ATP synthase is a ubiquitous rotary motor enzyme involved in cellular bioenergetics in eukaryotes and bacteria. It couples proton transport through a transmembrane complex (F_O_) with hydrolysis/synthesis of ATP on a peripheral catalytic complex (F_1_)^1–3^. In eukaryotes and photosynthetic or respiratory bacteria, F_O_F_1_ functions primarily to synthesize ATP. In contrast, many anaerobic bacteria require F_O_F_1_ to work as an ATP-driven proton pump to generate the cell’s membrane potential (Δψ) and help maintain pH homeostasis^4^; this reverse function is critical even for some strongly aerobic bacteria like *Pseudomonas aeruginosa* under fermentative conditions^5^. Despite general conservation of structure and function with mitochondrial F_O_F_1_ (mitoF_O_F_1_), bacterial F_O_F_1_ is now recognized as a promising target in the fight against multidrug-resistant (MDR) pathogens^6^, as bedaquiline (BDQ) has become a key part of front-line therapy for MDR tuberculosis^7^. The antimycobacterial activity of BDQ is mainly due to its interaction with ***c***-subunits of F_O_^8^. Each ***c***-subunit has a conserved acidic residue involved in proton transport, and a ring of ***c***-subunits spans the membrane; BDQ binds at multiple sites on the ***c***-ring, close to the essential carboxylates^9^. BDQ’s bactericidal action correlates with dramatic depletion of cellular ATP^10^, but may also involve its ability to collapse transmembrane ΔpH through its interactions with F_O_^11^.

It is increasingly apparent that bioenergetic factors are promising targets for antibiotic development^6^ and that a bacterium’s metabolic state can greatly impact the efficacy of existing antibiotics^12^. Thus, compounds targeting bacterial F_O_F_1_ may lead to new antibiotics^13^ and/or adjuvants that enhance the efficacy of other antibiotics^14^. Genetic knockout of F_O_F_1_ in *Escherichia coli*^15^ and *Staphylococcus aureus*^16^ enhances their sensitivity to several antibiotics. Several antifungal macrolides, including oligomycins and venturicidins, target F_O_ in membrane preparations isolated from mitochondria, chloroplasts, and bacteria. Mutations that make the enzyme resistant to these macrolides indicate that they bind at overlapping sites near the essential carboxylate on ***c***-subunits of F_O_ to block proton transport and thus inhibit ATP synthesis and hydrolysis on coupled F_1_^17^. The structural binding site for oligomycin on F_O_ has been determined recently and overlaps with the BDQ binding site noted above^18^. Oligomycin A is too toxic for clinical use but can act as a potent adjuvant for polymyxin B action against *S. aureus*^16^. Venturicidins have minimal toxicity in mice and dogs^19,20^; toxicity is minimal for some human cell lines but significant for others^21,22^. Alone, venturicidins do not exhibit antibacterial activity^21–23^. Recently, however, venturicidin A (ventA) was found to potentiate the action of aminoglycoside antibiotics against various MDR bacterial pathogens^22^; this adjuvant activity was suggested to be due to ventA’s direct inhibition of ATP synthesis by F_O_F_1_ and the subsequent increase in PMF, which should potentiate uptake of aminoglycosides. In particular, high concentrations of ventA dramatically enhanced bactericidal effects of gentamicin on *S. aureus* (MRSA) strains; reduction of cellular ATP content was considered a contributing factor, and was attributed to inhibition of F_O_F_1_. However, in the complex growth medium used, *S. aureus* can produce substantial ATP through substrate-level phosphorylation^24,25^ and F_O_F_1_ is not essential for growth^16,26^.

In this study, we report novel aspects of the interactions of venturicidins A and B (ventB) with F_O_F_1_-ATPase in inverted membrane vesicles from *E. coli* and *P. aeruginosa*. Adding ventA or ventB to membranes induces immediate inhibition of ATP hydrolysis that, at higher inhibitor concentrations, is followed by a time-dependent increase in ATPase activity. We show that the latter phase of ATPase recovery results from venturicidin-induced functional decoupling of F_1_-ATPase activity from the proton–transporting F_O_. Further, with *E. coli* membranes, we show that minimizing the fraction of MgADP-inhibited enzyme significantly increases the enzyme’s affinity for ventA and ventB. We discuss how these findings provide new insights into the likely mechanisms of venturicidins’ adjuvant activity for some antibiotics.

## Results

### Venturicidins exhibit complex, time-dependent effects on the rate of ATP hydrolysis by *E. coli﻿* membranes

**Figure 1:**
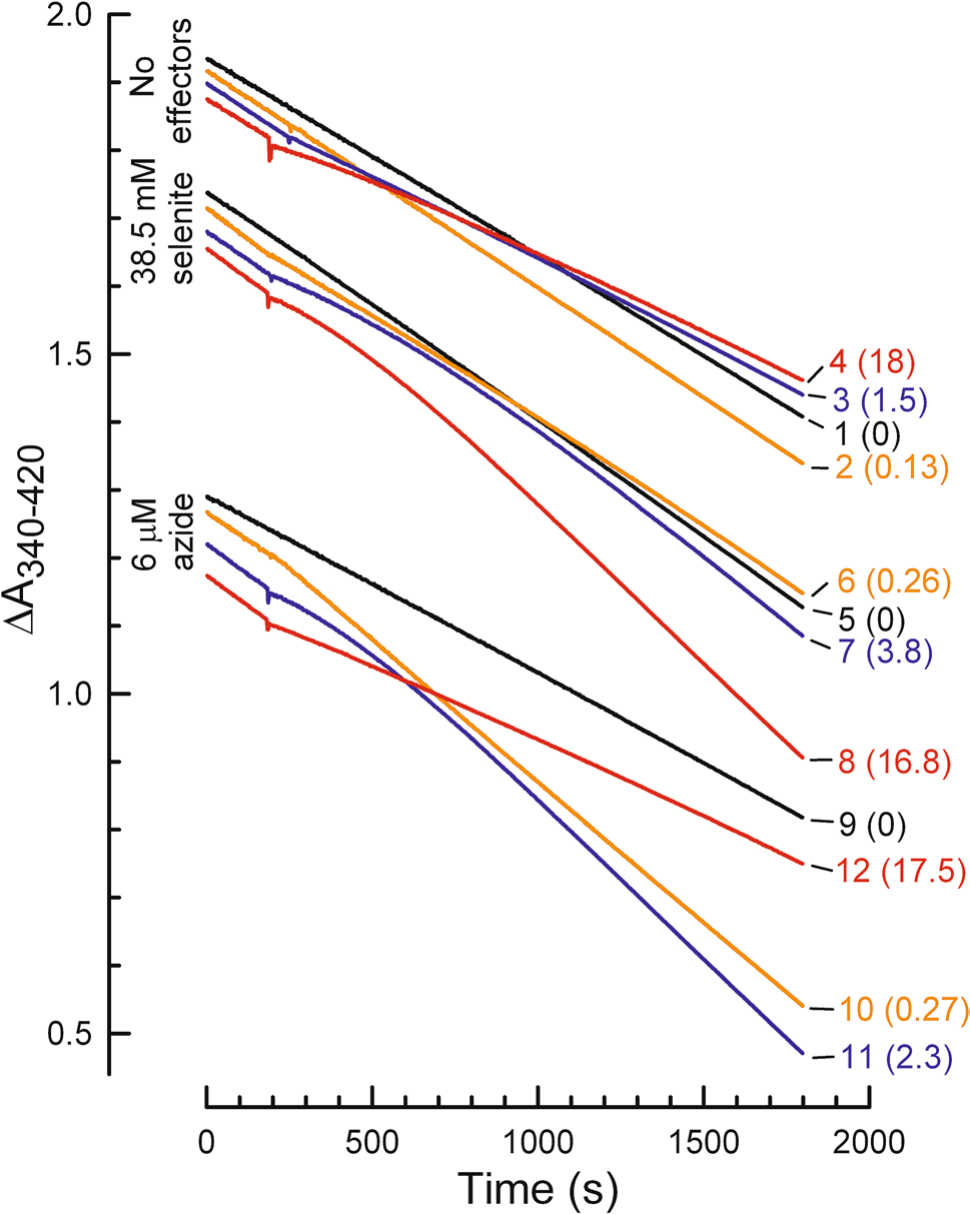
Venturicidin A has complex effects on ATPase kinetics of WT *E. coli* membranes**.** ATP hydrolysis was assayed as described in “Methods”. As noted beside the vertical axis, an additional effector was present initially for traces 5–8 (38.5 mM selenite) and 9–12 (6 µM azide). At 200 or 300 s, ventA was added to some assays; for each numbered trace, the final ventA concentration (μM) is in parentheses on the right, in the same color as the trace. Membrane protein per 1 ml assay: 3.36 µg (*traces 1–4*), 1.47 µg (*traces 5–8*) or 7.35 µg (*traces 9–12*). For each set of assay conditions (± selenite or azide), the specific ATPase activity (U/mg membrane protein) before adding ventA is listed in Table 1 under “100% ATPase”.

Figure 1 shows examples for the spectrophotometric assay used to monitor continuous hydrolysis of ATP by F_O_F_1_ on isolated membranes. ATP hydrolysis is enzyme-coupled to conversion of NADH to NAD^+^, which results in decreased light absorbance; thus, a larger rate of ATP hydrolysis is indicated by a steeper downward slope. The system also rapidly regenerates ATP, minimizing the concentration of product ADP during the assay. Unless noted, assays were preincubated to establish a steady state hydrolysis rate (see “Methods”) before the ‘zero’ point of measurements. Thus, control assays for each condition in Fig. 1 (traces 1, 5, 9) are essentially linear over the entire measurement period. With this method, we observed that ventA has complex effects on the kinetics of F_O_F_1_-ATPase activity of wild type (WT) *E. coli* membranes. Adding ventA at lower concentrations causes an immediate, small increase in the ATPase rate (Fig. 1, *trace 2*, + 11%, first minute post-addition), whereas an intermediate concentration immediately inhibits the rate (*trace 3*, –28%). At higher ventA concentrations, immediate inhibition is followed by time-dependent partial recovery of activity (*trace 4*) and, after completion of the recovery phase, a later ventA addition does not induce transient inhibition or further increase of the elevated rate (not shown). Immediate inhibition of ATPase activity by venturicidin has been reported for membranes from another bacterium, *Paracoccus denitrificans*^27^ but, for *E. coli*, assays typically included preincubating membranes with venturicidin (10–45 min) before adding ATP to start hydrolysis^23,28^. In the present study, effects of ventA on ATPase kinetics were similar when assays contained an optimal concentration of the activating anion selenite (*traces 6–8*), except that no increase in ATPase rate occurred at low concentrations of ventA. Similar effects of ventA were seen when assays contained the F_1_ inhibitor azide at a concentration that reduced the initial ATPase rate by ~ 50% (*traces 9–12*).

**Figure 2:**
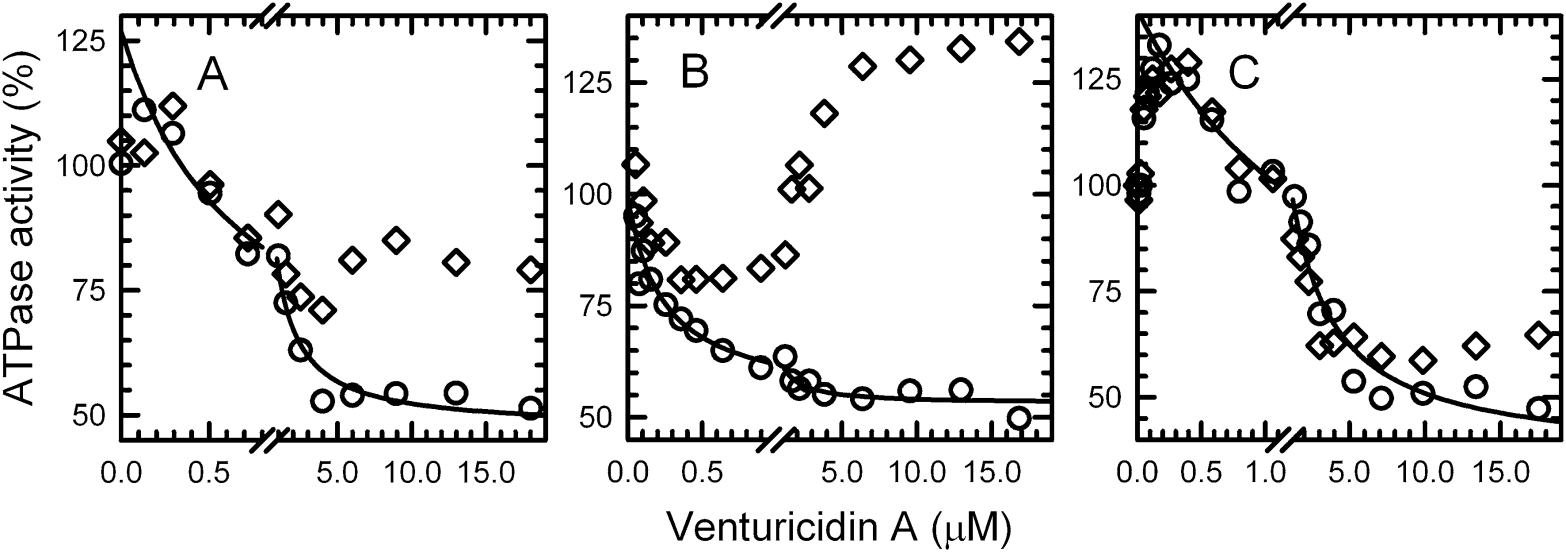
Concentration-dependence of venturicidin A’s immediate and time-dependent effects on the ATPase rate of WT *E. coli* membranes. WT membranes were assayed as in Fig. 1 and ATPase rates were measured ‘early’ (within 1 min after adding ventA, ○) and ‘late’ (during last 5 min of 30-min assay, ◊) in the absence (**A**) or presence of 38.5 mM selenite (**B**) or 6 μM azide (**C**). In each panel, the line shows the best fit of a hyperbolic equation, y = y_0_ + y_1_/(1 + x/K_i_), to the range of early rates (○*, y*) that decrease with increasing ventA concentration (x); this includes all points with [ventA] > 0 for panel (**A**), all points for panel (**B**), and points with > 0.15 μM ventA for panel (**C**). The activity (U/mg) corresponding to 100% for each condition and the best fit parameters for each curve are in Table 1.

The complex dependence of ATPase rates on ventA concentration is illustrated in Fig. 2 by comparing rates measured within 1 min after adding ventA (‘early’, ○) and during the last 5 min of each 30-min assay (‘late’, ◊). Very low concentrations of ventA (< 1 μM, Fig. 2A) immediately increase the early hydrolysis rate, which then remains nearly constant for the remaining assay period (*i.e.*, late rate is similar). Interestingly, this effect is absent in the presence of selenite (Fig. 2B) but is more pronounced in the presence of azide (Fig. 2C). At higher ventA concentrations, late rates deviate and become increasingly larger than early rates. Notably, the impacts of selenite and azide on the increasing late hydrolysis rates are opposite to their effects on early rates with low ventA: the time-dependent increase in late hydrolysis rates is enhanced by selenite and reduced by azide; the possible significance of these distinct impacts is addressed in the Discussion. The main point is that such a recovery phase in the action of venturicidins on F_O_F_1_-ATPases has not been reported before and would not have been detected in most earlier studies of venturicidin inhibition with *E. coli* membranes, which used end-point assays of ATPase over 3–6 min following addition of substrate ATP^23,28^.

Focusing on the early ATPase rates of Fig. 2A and starting from the lowest ventA concentration that yielded the largest early rate, inhibition by venturicidin fits well to a hyperbolic equation. Although the forms of venturicidin used in some early studies were not specified (A, B, or a mixture), the half-maximal inhibitory concentration of venturicidin that can be obtained from the data of ^28^ is comparable to the K_i_ of 0.7 μM obtained here (Table 1) under similar assay conditions (excess of Mg^2+^ over ATP); the results of ^23^ yield a larger value, near 7 μM, which could be due to more divergent assay conditions and/or a predominance of ventB in that venturicidin sample (see Supplementary Fig. S1).

**Table 1:** Best-fit parameters for early inhibition of *E. coli* membrane ATPase by venturicidin A.

Membranes, condition	100% ATPase (U/mg)	VentA-sensitive ATPase (y_1_)	ATPase at saturating ventA (y_0_)	VentA at half-max. inhibition (K_i_, μM)
WT, alone (Fig. 2A)	0.83 (± 0.07)	80% (± 5)	47% (± 2)	0.7 (± 0.1)
WT + selenite (Fig. 2B)	2.3 (± 0.2)	46% (± 2)	53% (± 1)	0.23 (± 0.04)
WT + azide (Fig. 2C)	0.42 (± 0.02)	106% (± 5)	36% (± 4)	1.7 (± 0.3)
ε88stop (Fig. 3A)	0.82 (± 0.05)	67% (± 4)	37% (± 3)	0.5 (± 0.1)
ε88stop + selenite (Fig. 3B)	3.4 (± 0.5)	66% (± 4)	37% (± 2)	0.09 (± 0.02)

**Figure 3:**
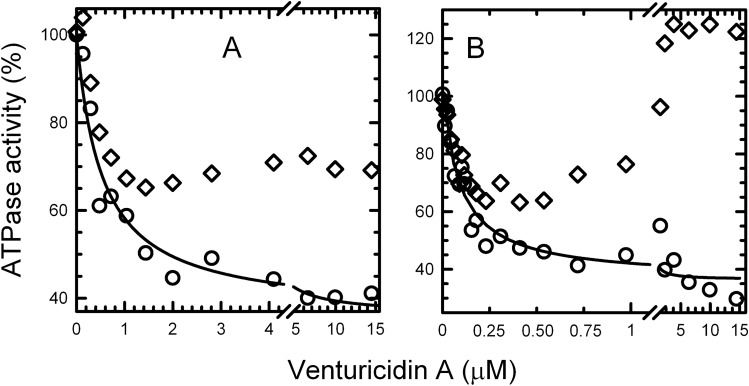
Concentration-dependence of venturicidin A’s effects on the ATPase rate of *E. coli* membranes with F_O_F_1_ lacking the ε subunit’s CTD. The ε88-stop membranes were assayed for ATPase as in Fig. 1, but with 3.31 μg (**A**) or 0.93 μg (**B**) of membrane protein. As in Fig. 2, rates were measured ‘early’ (○) and ‘late’ (◊) after adding ventA in the absence (**A**) or presence of 38.5 mM selenite (**B**). Each line shows the best fit of all early rates (○) to the equation shown for Fig. 2. Activities corresponding to 100% and the best fit parameters for the lines are listed in Table 1.

Values for the best-fit parameters are listed for curves of Figs. 2 and 3 for nonlinear regression of the hyperbolic dependence [y = y_0_ + y_1_/(1 + x/K_i_)] of relative ATPase early rates (y) on the concentration of ventA added (x). For each experiment, the 100% ATPase value is the average specific activity of all assays measured just before the addition of ventA.

### Venturicidins A and B exhibit selective affinity for active states of *﻿E. coli* F_O_F_1_-ATPase

As shown recently for assay conditions as in Fig. 2A without ventA, the measured ATPase rate for *E. coli* membranes actually reflects only ~ 20% of *E. coli* F_O_F_1_ complexes (EcF_O_F_1_) that are active on average, with most in transiently inactive states due to distinct actions of the ε subunit (≥ 50%) or inhibitory MgADP bound at one of the 3 catalytic sites on F_1_ (~ 30%)^29^. Certain anions stimulate ATPase activity of F_1_-ATPases^30,31^ including EcF_1_^32,33^ by decreasing the MgADP-inhibited fraction of the enzyme population^34^, most likely due to accelerated dissociation of inhibitory MgADP^35,36^. Selenite is one of the most potent anion-activators^31^, and the presence of optimal selenite stimulates ATPase activity of WT membranes 2.8-fold (Table 1, ‘100%’ value). At the same time, selenite reduces the *K*_i_ value for early inhibition by ventA by threefold (Fig. 2B, Table 1). This suggests that ventA has a higher affinity for the enzyme actively hydrolyzing ATP than for MgADP-inhibited EcF_O_F_1_. If so, then increasing the fraction of MgADP-inhibited EcF_O_F_1_ should increase the *K*_i_ value for ventA. To test this, we measured inhibition by ventA in the presence of azide, a well-known inhibitor of F-ATPases from all sources. Azide inhibits F-ATPase by increasing the fraction of MgADP-inhibited enzyme^34,37,38^. Including 6 µM azide in the assay (Fig. 2C) inhibits ATPase activity ~ 50% and increases the early *K*_i_ value for ventA by 2.4-fold (Table 1), further supporting the suggestion that the active form of EcF_O_F_1_ has higher affinity for ventA than does the MgADP-inhibited form. In a prior study with membranes from *P. denitrificans*, ATPase activity was ~ threefold more sensitive to ventA inhibition when F_O_F_1_ was activated by the oxyanion sulfite than when it was activated by PMF, and that was interpreted as indicating two distinct active states^27^. However, consistent with our results, the ATPase activity was also increased 2- to fourfold more by sulfite than by PMF, which could indicate sulfite was simply more effective, driving a larger fraction of F_O_F_1_ out of the MgADP-inhibited state.

We next investigated whether the ε-inhibited state of EcF_O_F_1_ also impacts interactions of ventA with the enzyme. The N-terminal domain (NTD) of ε is required for F_O_F_1_ assembly and functional coupling of F_1_ to F_O_ in eukaryotic and bacterial species; the C-terminal domain (CTD) of ε can auto-inhibit F_O_F_1_ and isolated F_1_ from some bacteria and from chloroplasts, but the mitochondrial homolog (δ) is not inhibitory^39,40^. Thus we measured the effects of ventA on ATPase activity of *E. coli* membranes expressing ε88-stop, which lacks the autoinhibitory εCTD (Fig. 3). From Table 1 (100% ATPase values), it is clear that selenite activates membrane ATPase more (fourfold) for ε88-stop than for WT (2.8-fold). This is because, without the inhibitory εCTD, more enzyme complexes shift to the MgADP-inhibited state, which can be activated by selenite^29^. Consistently, the results of Fig. 3 show that the *K*_i_ for early inhibition of ε88-stop membranes by ventA is fivefold lower in the presence of selenite than in its absence (Table 1). This further supports the suggestion that ventA binding differentiates between MgADP-inhibited and active forms of EcF_O_F_1_. In contrast, early inhibition of ATPase by ventA has similar *K*_i_ values for WT and ε88-stop membranes in the absence (0.7 *vs* 0.5 μM) or in the presence of selenite (0.23 *vs* 0.09 μM). This suggests that the εCTD-inhibited state has less impact on the enzyme’s affinity for ventA than does the MgADP-inhibited state. Also, the slow ATPase recovery period induced by higher concentrations of ventA is similar for membranes with WT ε (Fig. 2A,B) or ε88stop (Fig. 3A,B), indicating that effect is not significantly altered by the presence of the εCTD.

As noted earlier, we also tested the effects of venturicidin B (ventB) on ATP hydrolysis by WT *E. coli* membranes (see Supplementary Fig. S1). Without selenite, immediate activation induced by low concentrations of ventB is moderate but more significant than that observed for ventA (Fig. 2A); also similar to ventA, higher ventB concentrations induce immediate inhibition and time-dependent recovery of ATPase activity. With selenite present, as for ventA (Fig. 2B), low concentrations of ventB do not induce immediate activation. With or without selenite present, the K_i_ for inhibition of the early rate by ventB (see Supplementary Fig. S1) is ≥ tenfold higher than the respective *K*_i_ values for ventA, indicating higher affinity of EcF_O_F_1_ for ventA than for ventB. However, similar to ventA, the K_i_ of ventB is reduced (2.3-fold) when MgADP-induced inhibition is relieved by selenite. Thus, both venturicidins A and B exhibit lower affinity for EcF_O_F_1_ that is in the MgADP-inhibited state.

### Venturicidin-induced recovery of ATPase activity is ATP–dependent

Time-dependent recovery of ATPase activity that follows immediate venturicidin-induced inhibition, reported here for the first time, occurs at higher venturicidin concentrations than required for initial inhibition. Thus it is likely that the venturicidin-binding site(s) that induce recovery of ATPase activity differ from the site(s) that cause initial inhibition. At high ventA concentrations, ATPase recovery is completed within ~ 20 min after adding ventA (*traces 4* and *8* in Fig. 1, and Fig. 2). In the original study of venturicidin’s inhibition of *E. coli* membrane ATPase^23^, membranes were incubated with venturicidin for 45 min before adding ATP and assaying ATPase for 3 min. The fact that they did not observe decreased inhibition at high venturicidin concentrations suggests that MgATP is required for the recovery phase to occur after venturicidin-induced inhibition. To test whether this is the case, we incubated WT *E. coli* membranes at 30 °C in the assay medium containing optimal selenite but lacking ATP, PEP, and NADH in the presence and absence of 10 μM ventA for 30 min and then started the ATPase assay by adding a mixture of ATP, PEP, and NADH. In the absence of ventA, preincubating membranes without ATP does not affect kinetics of ATP hydrolysis significantly (see Supplementary Fig. S2, *trace 1*). If ATP were not required for the relatively fast recovery of ATPase activity after ventA-induced inhibition, then ventA-induced inhibition should not be observed after a 30-min incubation with ventA but without ATP. Supplementary Fig. S2 shows that this is not the case: preincubation with ventA decreases the initial ATPase rate by about 40%, which is similar to the extent of immediate inhibition by ventA under the same assay conditions (Fig. 2B). The recovery of activity to 130% after preincubation with ventA and subsequent addition of ATP (see Supplementary Fig. S2, *trace 2*) is also similar to that observed when ventA was added to membranes already hydrolyzing ATP (Fig. 2B). Thus, the time-dependent recovery of activity observed after ventA-induced inhibition requires ATP and likely catalytic turnover.

### Venturicidin-induced recovery of ATPase activity involves functional decoupling of F_1_-ATPase from membrane-embedded F_O_

**Figure 4:**
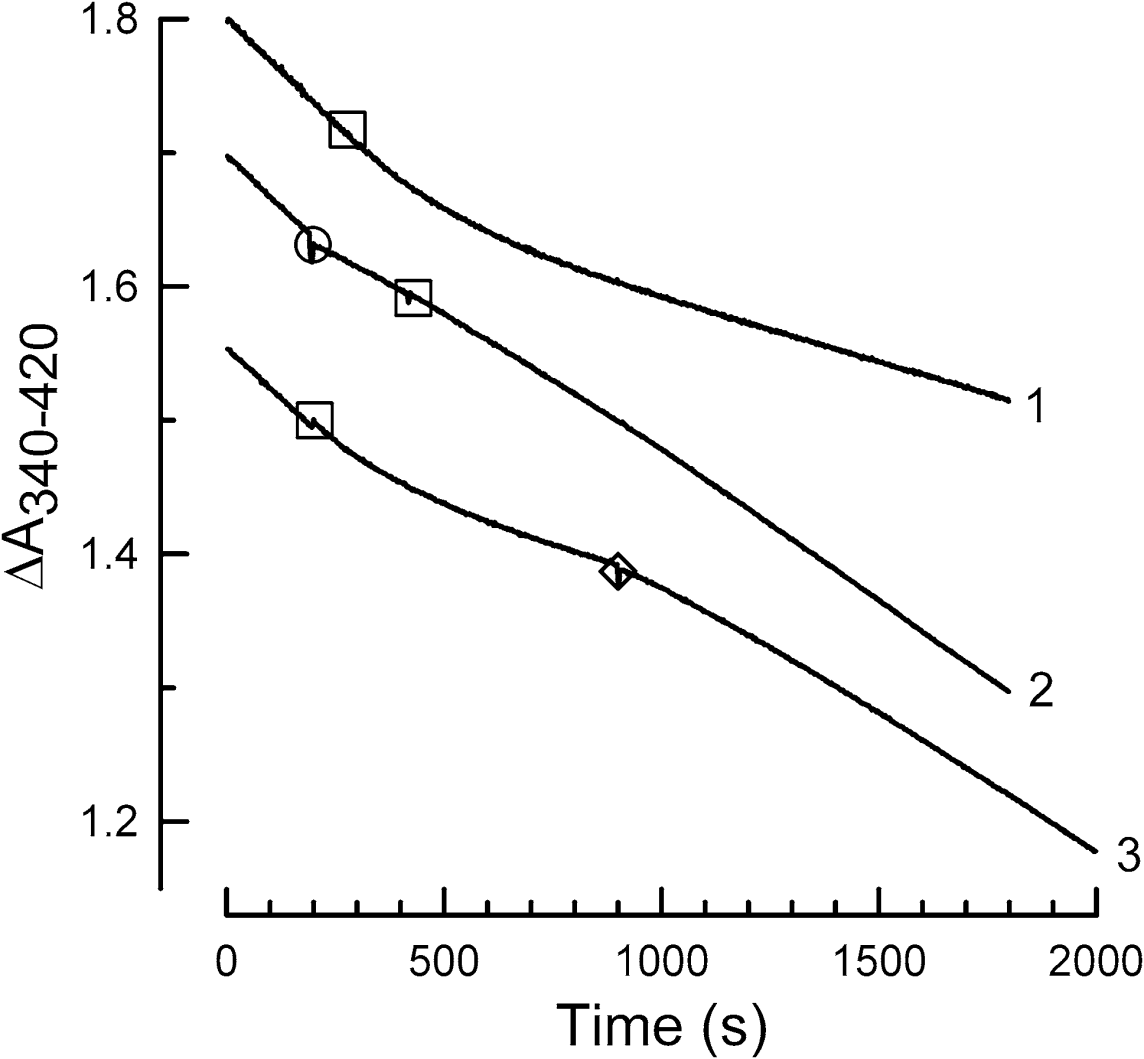
VentA-induced increase in ATPase activity is not blocked by DCCD modification of F_O_. ATP hydrolysis was assayed as in “Methods”, using 3.36 µg of WT *E. coli* membrane protein. The mean of ATPase activity during the first 3 min of all assays is 0.83 (± 0.02) U/mg. DCCD was added to each assay at 0.1 mM (□) and ventA was added either before DCCD (○**, ***trace 2*, 10 μM ventA) or after DCCD (◊, *trace 3*, 15 μM ventA). During the last 3 min of assays, ATPase activity (± SD, n = 3) is 0.20 (± 0.02), 0.53 (± 0.05), or 0.49 (± 0.06) U/mg for conditions of representative *traces 1–3*, respectively.

Since ventA targets F_O_ within the membrane^23^, the ventA-induced, time-dependent recovery of ATPase activity could indicate that the EcF_1_-ATPase is functionally decoupling from EcF_o_. We tested this using DCCD (N,N′-dicyclohexylcarbodiimide), a well-known inhibitor of F_O_F_1_-ATPases that acts by covalently modifying ***c*** subunits of F_O_ on a conserved acidic residue that is essential for proton transport^41^. Figure 4 shows that, within 10 min of adding DCCD, ATPase activity of WT membranes is inhibited by 73% (trace 1). Adding DCCD after 15 μM ventA (*trace 2*) does not induce inhibition and fails to prevent time-dependent recovery of ATPase activity, suggesting the recovery phase of ventA action is due to EcF_1_ that is no longer functionally coupled to EcF_O_. However, since an early study reported that venturicidin slows labeling of ***c*** subunits on EcF_O_ by [^14^C]DCCD, at least in membranes stripped of EcF_1_^23^, we investigated how ventA would affect ATPase activity of membranes that had been preincubated with DCCD. As shown by *trace 3*, adding 10 μM ventA to DCCD-inhibited membranes induces a time-dependent increase of ATPase activity from 37 to 70% of the activity observed before adding DCCD, analogous to the recovery phase observed after initial inhibition induced by high concentrations of ventA (Fig. 1, *traces 4* and *8*). This does not conflict with the ATP-dependence of ATPase recovery, as it has been shown that ATP can drive partial rotation of the ***c***-ring in EcF_O_F_1_ that has been inhibited by [^14^C]-DCCD^42^. Results similar to those in Fig. 4 were also obtained in the presence of selenite (see Supplementary Fig. S3). Since DCCD inhibition involves irreversible covalent modification of ***c***-subunit(s), the recovery phase induced by ventA must be due to decoupling of F_1_-ATPase from F_O_.

**Figure 5:**
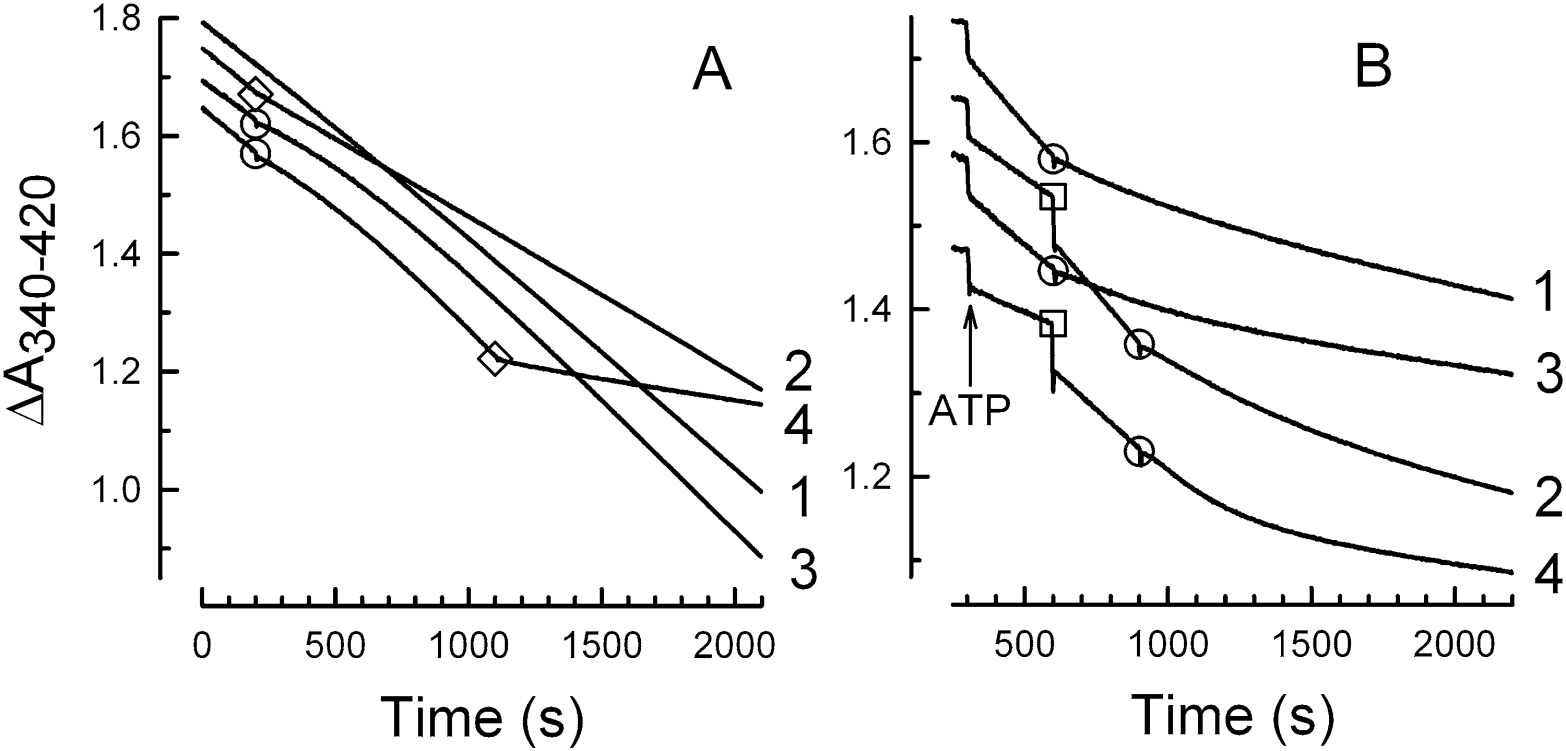
The ventA-induced increase in ATPase activity is inhibited by adding excess ε subunit. Panel (**A**): ATPase was assayed as described in “Methods” using 1.47 µg of WT membrane protein in the presence of 38.5 mM selenite. Symbols indicate addition of 67 nM ε (◊) or 9.5 μM ventA (○). Early rate (U/mg, first 3 min of assay): 2.30 (SD, ± 0.13 for traces 1–4). Late rates (U/mg, last 3 min of assay): *#1*, 2.2 (± 0.3, n = 3); *#2*, 1.4 (± 0.2, n = 3); *#3*, 2.7 (± 0.3, n = 3), *#4*, 0.28 (± 0.05, n = 3). For *trace #4*, during 100 s immediately before adding ε, the rate is 2.5 (± 0.4, n = 3) U/mg. Panel (**B**): traces representative of 2 experiments in which membranes were first added to the assay medium (see “Methods”) but lacking ATP and containing 88 nM ε. Membranes were either WT (traces: #1, 4.2 μg; #2, 2.1 μg), or ε88-stop (traces: #3, 3.1 μg; #4, 1.24 μg). Arrow indicates addition of 1 mM ATP to initiate each assay. Symbols indicate addition of 38.5 mM selenite (□) or 10 μM ventA (○).

In vitro, exposing inverted *E. coli* membranes to low ionic strength and chelators of divalent metals induces dissociation of EcF_1_ from the membrane as a soluble ATPase. Re-binding EcF_1_ to EcF_O_ in EcF_1_-depleted membranes and coupling of EcF_1_-ATPase activity to proton translocation through F_O_ require the presence of both δ and ε subunits^43^. Therefore, decoupling induced by high concentrations of ventA may be due to disrupting F_1_-F_O_ interactions involving ε and/or δ, which leads to dissociation of EcF_1_ from EcF_O_. Since δ does not affect EcF_1_-ATPase activity but bound ε inhibits it^44,45^, we examined whether ATPase activity recovered after ventA action is a result of relieving inhibition by ε, which can dissociate from soluble EcF_1_^46^. Thus we tested the effect of exogenous ε on the ATPase recovery induced by higher ventA concentrations (Fig. 5). Without ventA, adding excess ε to WT membranes hydrolyzing ATP in the presence of selenite leads to an immediate ~ 30% inhibition of ATPase activity (Fig. 5A, *trace 2 vs 1*). This suggests the slow increase in activity observed during initial ATP hydrolysis (~ 25%, as noted in Materials and Methods) occurs at least in part because a portion of the enzyme has lost inhibition by endogenous ε. Adding 9.5 μM ventA first (Fig. 5A, *traces 3*, *4*) induces immediate inhibition followed by time-dependent recovery, and subsequent addition of excess ε immediately inhibits that ATPase activity by ~ 90% (*trace 4*), as occurs with soluble EcF_1_^46^. Consequently, adding excess ε before ventA should eliminate the recovery phase. This was confirmed by assays done without the usual hydrolysis preincubation (Fig. 5B). With excess ε already present with membranes, hydrolysis was initiated by adding ATP (Fig. 5 B, arrow). Adding 10 μM ventA next immediately inhibited WT ATPase activity 55–60% in the absence (*trace 1*) or presence of selenite (*trace 2*) but without subsequent activation; instead, additional time-dependent inhibition of ~ 25% occurred. Similar results were obtained with ε88-stop membranes (Fig. 5B, *traces 3*, *4*). Note that, in Fig. 5A, the slow ventA-induced recovery phase is complete before addition of excess WT ε (trace 4 rate is similar to final rate for trace 3), and maximum inhibition by ε occurs immediately. Thus, the same ventA-dependent limiting step is likely responsible for the slow inhibition observed with excess ε already present in the assays of Fig. 5B. This is supported by further assays without preincubation with ATP: once ventA-dependent recovery of activity is nearly complete, adding excess WT ε induces strong immediate inhibition without a significant time-dependent component, for both WT and ε88-stop membranes (see Supplementary Fig. S4).

### Venturicidin-induced functional decoupling also occurs in F_O_F_1_ of *﻿P. aeruginosa*

**Figure 6:**
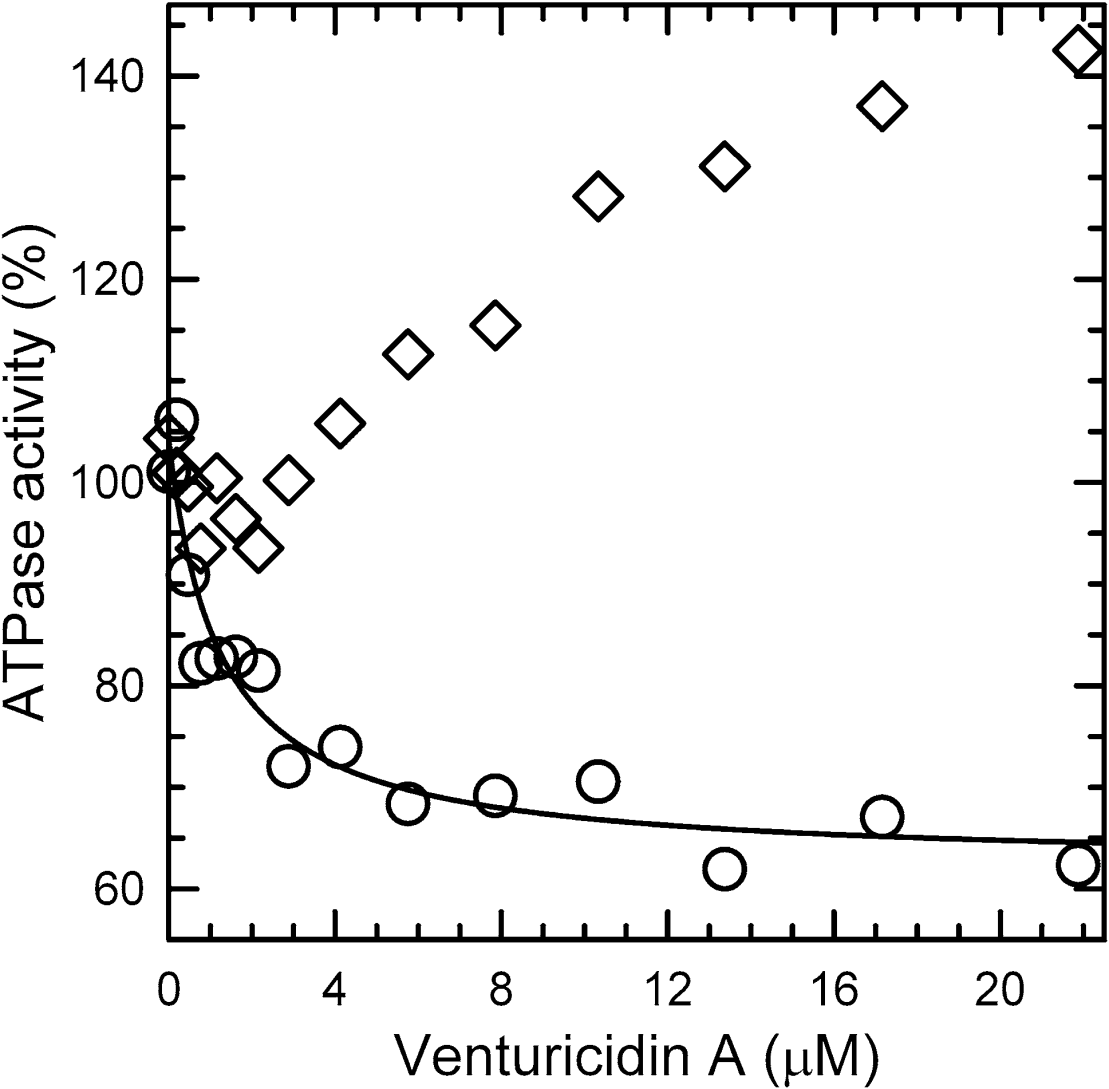
Complex effects of venturicidin A on ATPase rate are also observed with membranes from *P. aeruginosa*. PAO1 membranes (6.72 μg) were assayed in the presence of 38.5 mM selenite as for Fig. 1 and ATPase rates were measured at early (○) and late (◊) periods after adding ventA, as described for Fig. 2. The rate before ventA addition (100%) is 0.45 U/mg, and inhibition of the early rate fits a hyperbolic dependence on ventA concentration (see equation, Fig. 2 legend) with a K_i_ = 1.2 μM (± 0.4) for 42% (± 3) ventA-sensitive ATPase and 62% (± 2) ATPase remaining at saturating ventA.

To investigate whether the complex effects of venturicidins are relevant to other species of pathogenic bacteria, we tested membranes from *P. aeruginosa* since, for three clinical isolates of aminoglycoside-resistant *P. aeruginosa*, ventA enhanced sensitivity to Gentamycin by 2-, 4- and 8-fold^22^. Compared to WT *E. coli* membranes (0.83 U/mg, Table 1), membranes from *P. aeruginosa* strain PAO1 exhibit low ATPase activity (~ 0.1 U/mg), but it is still attributable to PaF_O_F_1_ since it can be inhibited 80–90% by excess azide. The PAO1 membrane ATPase could also be activated ~ fourfold by selenite, indicating PaF_O_F_1_ is latent at least in part due to MgADP inhibition. Because of the low intrinsic ATPase activity of PAO1 membranes, we tested the effects of ventA with selenite present, and the results are very similar to those for *E. coli* membranes with selenite present (Figs. 2B, 3B, Table 1). VentA inhibits the early ATPase rate of PAO1 membranes (Fig. 6, ○) up to 42% and the late rate shows concentration-dependent recovery of activity up to 140% of the uninhibited rate. This indicates that PaF_O_F_1_ is also sensitive to the capacity of higher venturicidin concentrations to functionally decouple F_1_-ATPase activity from membrane-embedded F_O_.

## Discussion

**Figure 7:**
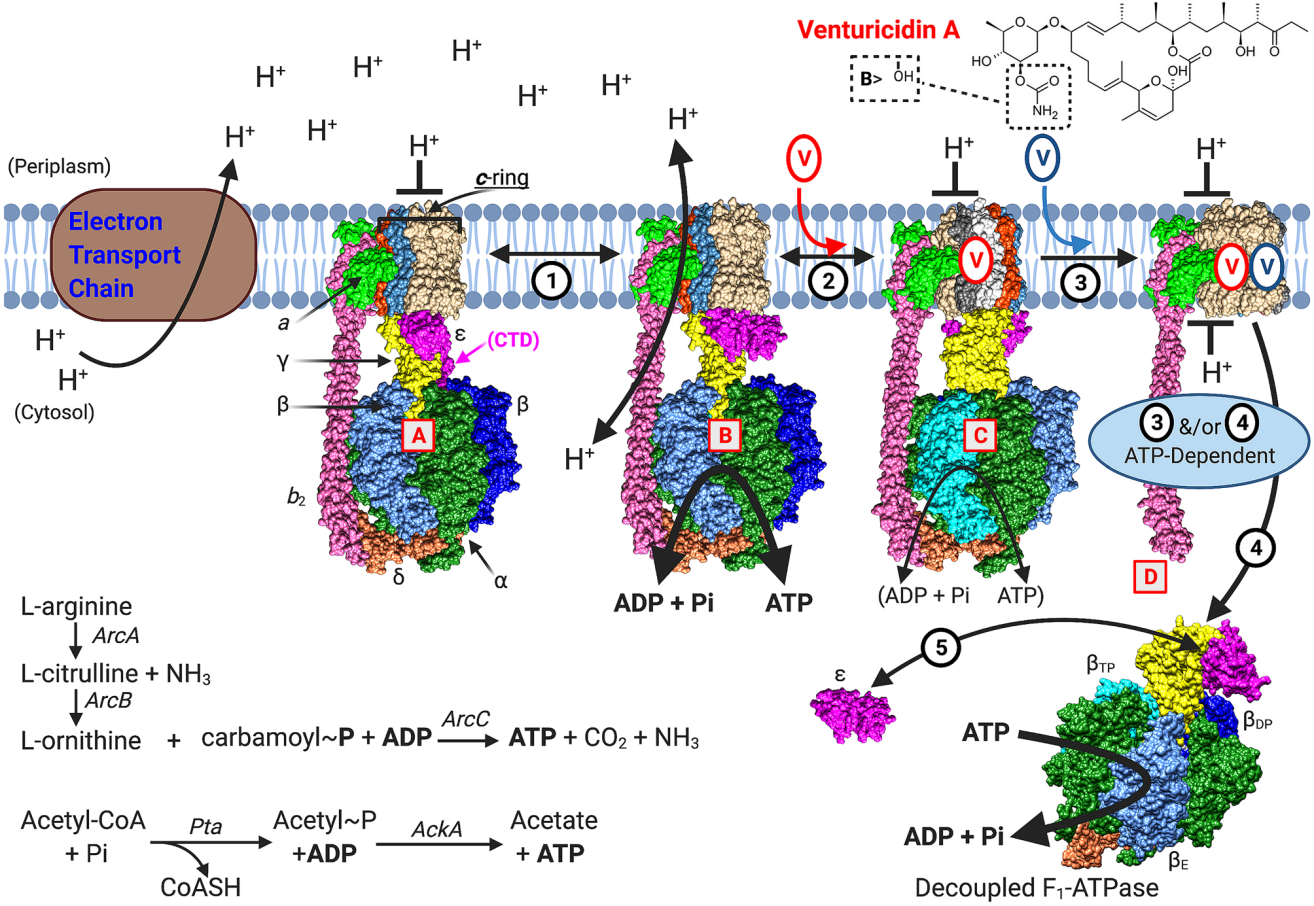
Multiple actions of venturicidins on bacterial F_O_F_1_, can enhance depletion of cellular ATP. In respiring bacteria, the electron transport chain pumps protons (H^+^) out of the cell to generate PMF; return flux of H^+^ through F_O_ of active F_O_F_1_ (**B**) drives synthesis of ATP at the 3 F_1_ catalytic sites. Active forms of F_O_F_1_ can convert (***1***) to transiently inactive states such as ε-inhibited (**A**) or MgADP-inhibited (not shown), and increased PMF favors return to the active state. Without respiration, active F_O_F_1_ can work in reverse as an ATPase-driven H^+^-pump to generate PMF, as long as sufficient cellular ATP is maintained by glycolysis or other pathways for substrate-level phosphorylation (*e.g.*, lower left^5,24,25^). Active F_O_F_1_ (**B**) has the highest affinity site(s) for venturicidin binding to F_O_ (***2***), which inhibits H^+^ transport and catalysis by coupled F_1_ (**C**). At higher concentrations, venturicidin binds (***3***) at additional sites and (***4***) induces functional decoupling of F_1_ (**D**), while venturicidin still blocks H^+^ transport through F_O_^23^. Decoupled F_1_ can only catalyze ATP hydrolysis; many F_1_ complexes may shift to the εCTD-inhibited state (not shown) but the portion of active F_1_-ATPases can increase if ε dissociates (K_D_ < 1 nM)^46^ (***5***). Thus, decoupled F_1_-ATPase may significantly deplete levels of cellular ATP. In F_O_, 4 subunits of the ***c***-ring are colored distinctly (steel blue, orange-red, light and dark grays) to indicate the major rotational states identified by Cryo-EM for *E. coli* F_O_F_1_^59^. In F_1_, the catalytic β subunits are different shades of blue to reflect their distinct conformations (all 3 βs are partially visible, labeled in **D**). One state (PDB: 3OQR) is shown for (**A**,**B**) and decoupled F_1_ (**D**). State (**C**) (PDB: 3WNQ) follows one 120° rotary step (1 ATP hydrolysis) and state (**D**) shows F_O_ after another 120° step (PDB: 6OQW). The compact, non-inhibitory conformation of ε (PDB: 1BSN) is shown in all states except (**A**). Molecular models were rendered with Chimera^60^; composite figure was prepared with BioRender.com.

We report multiple effects of venturicidins on F_O_F_1_ in bacterial membranes, and these provide insights that could improve the efficacy of targeting bacterial F_O_F_1_ for developing new antibiotics and/or adjuvants for existing antibiotics. Figure 7 provides a schematic overview. First, venturicidins have higher affinity for F_O_ when the coupled F_1_ is in an active state (Fig. 7, B) rather than shifted (*step 1*) to transiently inactive but significantly populated forms, the MgADP-inhibited or εCTD-inhibited states (Fig. 7, A). As evident from results summarized in Table 1, removing the εCTD has a small effect on the sensitivity of EcF_O_F_1_ to immediate inhibition by ventA, but the MgADP-inhibited state has greater impact. For example, when the proportion of WT EcF_O_F_1_ in the MgADP-inhibited state is minimized by selenite, ATPase activity is > fivefold higher and ~ sevenfold more sensitive to immediate inhibition by ventA than when the fraction of MgADP-inhibited complexes is increased by azide. Selectivity is also indicated in the modest activation of ATPase with sub-inhibitory concentrations of ventA (Fig. 2A) or ventB (see Supplementary Fig. S1): activation is eliminated by selenite (Fig. 2B, Supplementary Fig. S1) but enhanced by azide (Fig. 2C). These different impacts of the MgADP– and εCTD–inhibited states suggest that the affinity of F_O_ for venturicidin is impacted by the rotational sub-states of F_1_: the orientation of γ’s central, asymmetric rotor shaft within the α_3_β_3_ assembly differs by ~ 30° for the εCTD-inhibited state of EcF_1_ *vs* the MgADP-inhibited state identified with mitoF_1_^46^. The enzyme’s rotary coupling mechanism^2,3^, with 120° rotation per ATP hydrolyzed or synthesized, involves smaller angular sub-steps, and a range of rotational orientations have been identified in F_1_ structures (*e.g*., Fig. 9 of ref. ^46^). Thus, further analyses of distinct rotational states of F_O_F_1_ from different species may help identify specific states with the highest affinity binding of inhibitors to F_O_ or F_1_, and/or sites that are most selective for binding to bacterial species of F_O_F_1_.

Our more surprising and novel finding is that, although initially inhibitory (Fig. 7, *step 2*), venturicidin at higher concentrations binds at additional sites (*step 3*) and induces time– and ATP–dependent recovery of ATPase activity (*steps 3, 4*). For *E. coli*, we showed that recovered activity is due to decoupling of F_1_-ATPase from venturicidin–inhibited F_O_ (Fig. 4). We then showed (Fig. 5) that decoupling is likely due to F_1_ dissociation from the membrane (Fig. 7, *step 4*). In our in vitro assays with dilute membranes, ε would dissociate from a significant portion of decoupled F_1_ (Fig. 7, *step 5*); this can explain why, with selenite present, recovered ATPase activity exceeded the activity present before adding venturicidin (Figs. 2B, 3B, and Supplementary Fig. S1). In assays without selenite (same Figs, panel A), ε dissociation would allow more decoupled F_1_ to become MgADP-inhibited^46^, resulting in lower levels of recovered activity. For growth of *E. coli* on glucose, decoupled cytosolic F_1_-ATPase lacking ε causes a greater growth defect than does a mutant completely lacking F_O_F_1_; however, the defect was reversed if ε was expressed with decoupled F_1_^47^. Thus, the impact of venturicidin-decoupled F_1_ on bacterial ATP content should depend on the concentration of dissociated F_1_ and the fraction of F_1_ complexes that are active due to dissociation of ε (Fig. 7, *step 5*) and/or the presence of endogenous activating oxyanions. The prominence of ε– and MgADP–inhibited states can vary between bacterial species^39^ but has not been studied for many pathogens; in some species, inhibition by the εCTD may be supplemented or superseded by a unique subunit^48^ or by a unique segment of another F_1_ subunit^49^.

Venturicidin’s overall impact on cellular ATP should depend on the bacterium’s environmental and metabolic limitations. This could explain the variable efficacy observed in a recent study that identified ventA as an adjuvant that potentiates aminoglycoside antibiotics against several MDR pathogens^22^. VentA showed minimal adjuvant action against *E. coli*, which is capable of rapid substrate-level phosphorylation in complex growth medium. In contrast, ventA enhanced gentamycin action up to eightfold against clinical isolates of *P. aeruginosa*, which is highly dependent on oxidative phosphorylation since it lacks the Embden–Meyerhof–Parnas glycolytic pathway. We report ventA-induced recovery of ATPase activity with *P. aeruginosa* membranes (Fig. 6), and its likely importance for venturicidin’s adjuvant effect is supported by an early study that showed distinct effects of venturicidin on *P. aeruginosa* in different growth conditions^50^. During respiration, venturicidin reduced cellular ATP ~ fivefold, increased the membrane potential (Δψ), and cells retained PMF-driven flagellar motility. Inhibition of F_O_F_1_ alone could explain those effects but not venturicidin’s effects during anaerobic fermentation. In that case, most cellular ATP would be produced by fermenting added L-arginine (Fig. 7, lower left) and PMF would be maintained by F_O_F_1_ acting as an ATPase–driven proton pump^5^. Compared to respiratory conditions, anaerobic Δψ and cellular ATP were lower but supported flagellar motility for 45 min; venturicidin decreased cellular ATP ~ tenfold and eliminated Δψ and flagellar motility within 3.5 minutes^50^. Such rapid depletion of cellular ATP would not be expected from inhibition of F_O_F_1_–ATPase. We propose that the high venturicidin concentration decoupled F_1_, which rapidly hydrolyzed ATP generated by limited substrate-level phosphorylation. Like *E. coli*, *S. aureus* is capable of substantial substrate-level phosphorylation in complex growth media without glucose^24,25^ (*e.g.*, Fig. 7, lower left). However, for several MRSA strains, ventA enhanced sensitivity to gentamycin 8- to 16-fold^22^, and some results suggest to us that ventA-induced decoupling of F_1_-ATPase is involved. For the MRSA strain tested further, the maximal effect on Δψ (by inhibiting F_O_) was achieved at 16 μg ventA/ml but cellular ATP was reduced by only ~ 40%; fourfold greater ventA was needed to reduce cellular ATP by ~ 90%^22^. Thus, the dual actions of ventA on F_O_F_1_ reported here may be important for optimal adjuvant efficacy of ventA against diverse bacterial pathogens.

Venturicidins inhibit F_O_F_1_-ATPase to varying extents in membranes from mitochondria, chloroplasts, and bacteria^17^ and, to our knowledge, the current study is novel in finding that higher venturicidin concentrations induce initial inhibition followed by time-dependent recovery of ATPase activity for membranes from two Gram-negative bacteria. One early study compared inhibitor sensitivities for mitoF_O_F_1_ in membranes *vs* detergent-solubilized mitoF_O_F_1_: oligomycin inhibited ATPase activity of both forms, whereas ventA inhibited membrane ATPase 95% but stimulated the solubilized ATPase up to 3-fold^51^. Thus, in membranes, mitoF_O_F_1_ likely resists decoupling due its more robust stator stalk and/or its usual dimeric state that is often disrupted by solubilization^2,3^. Nevertheless, this suggests a common mechanism for potential decoupling of F_1_-ATPase from F_O_. Results of a recent study suggest that decoupling F_O_F_1_ by an F_O_-targeted inhibitor is not unique to venturicidins: detergent-solubilized F_O_F_1_ from *M. smegmatis* (MsF_O_F_1_) was inhibited ~ 80% by nanomolar BDQ but most activity was restored by micromolar BDQ, although time dependence was not noted^49^. Recovery of ATPase activity by micromolar BDQ has not yet been observed with mycobacterial membranes [*e.g.*, with 5 min assays^52^) but this could indicate BDQ-decoupled F_1_-ATPase activity contributes significantly to BDQ’s antibiotic efficacy: low (nM) BDQ is rapidly bacteriostatic for *M. tuberculosis* cultures but slow bactericidal action is greatly enhanced by higher (μM) BDQ, which also dramatically depletes cellular ATP^10^. The recent cryo-EM study^49^ determined high-resolution structures of MsF_O_F_1_ ± BDQ, with distinct high affinity sites at the “leading” and “lagging” interfaces of the ***c***-ring with subunit ***a***, and 5 lower affinity sites on ***c***-subunits not contacting ***a***. This is likely the case for different affinity sites noted here for the dual effects of venturicidins. For MsF_O_F_1_ incubated with excess BDQ, cryo-EM did not show decoupled complexes but ATP was absent, consistent with our finding that decoupling is ATP-dependent. A likely scenario is that decoupling of BDQ-saturated F_O_F_1_ involves the added stress of partial rotation driven by ATP binding on F_1_.

## Methods

Venturicidin A was from BioViotica, and venturicidin B was from Cayman Chemical. Lactate dehydrogenase (salt-free, lyophilized) and Pyruvate kinase (type II) were both rabbit muscle enzymes obtained from Sigma-Aldrich. Before use, pyruvate kinase was desalted by column centrifugation^53^.

Plasmid pXH302S, which encodes the WT *atpC* gene and expresses near haploid levels of the ε subunit^54^, was subjected to fusion PCR mutagenesis to construct plasmid pMBε1, with an N-terminal affinity tag (MHHHHHHGH) added prior to the initial Met residue. To express the ε88-stop subunit, an AfeI-ScaI restriction fragment of WT pMBε1 was replaced by the analogous fragment from pH_6_ε88-stop^46^. To express haploid levels of EcF_O_F_1_ containing WT-ε or ε88-stop, the appropriate version of pMBε1 was transformed into *E. coli* strain XH1, which has a chromosomal deletion of the *atpC* gene for ε^54^. For these transformed strains, cultures were grown and inverted membrane vesicles (IMV) were prepared according to ^55^. WT ε, with the same N-terminal His_6_-tag, was over-expressed from pH_6_ε and purified as described^46^.

*P. aeruginosa* strain PAO1 was grown in LB medium at 37 °C as described^56^. For preparation of IMV, final cultures used 1 L of LB per 2L baffled flask and were inoculated from an overnight culture at an initial OD_600_ ~ 0.025. Cultures were shaken at 250 rpm and monitored until they reached mid- to late-logarithmic growth phase (OD_600_, 1–1.4). Cultures were chilled and subsequent steps for cell lysis and isolation of IMV were done at 4ºC. Cells were sedimented by centrifugation (3,700 × *g*, 30 min). Cells from 1 to 2 L of culture (~ 2–3 g wet cells/L) were resuspended in 25–40 ml of STEM buffer (TEM buffer plus sucrose^55^), transferred to a 50 ml Oak Ridge tube, sedimented again (11,617 × *g*, 10 min), resuspended in 20–40 ml of STEM and frozen at -80 °C. Thawed cells were lysed by two sequential passages through an SLM-Aminco French pressure cell at ~ 16,000 psi, essentially as described for *E. coli*^55^; the pressure cell was enclosed in a clear biohazard bag (MiniGrip, IP2024B3T) during use to contain any aerosols formed. Unlysed cells and cell wall debris were removed by centrifugation (50 ml Oak Ridge tubes, 16,743 × *g*, 25 min): the supernate was diluted with MTGM7.5 buffer (50 mM MOPS-TRIS, 10%(v/v) glycerol, 5 mM Mg(CH_3_COO)_2_, pH 7.5) and centrifugation was repeated at least twice, recovering the supernate each time. The final supernatant lysate was stored at -80 °C. To isolate IMV, the cleared lysate was thawed quickly and diluted with MTGM7.5 as needed to fill 1 or 2 polycarbonate tubes for use in a Beckman Ti70 rotor. The IMV (membranes) were sedimented by ultracentrifugation (250,000 × *g* max) for 1 h, the membranes were resuspended in MTGM7.5 buffer to fill one Ti70 tube and ultracentrifugation was repeated for 45 min. The membranes were resuspended in a small volume of MTGM7.5 buffer (typically ≥ 10 mg membrane protein/ml) and homogenized manually (Potter–Elvehjem type). Small aliquots of the final membrane sample were quick-frozen in liquid nitrogen and stored at − 80 °C.

ATP hydrolysis by membrane vesicles was assayed spectrophotometrically^57^ at 30 °C using an open-chamber, diode array spectrophotometer (Hewlett Packard 8453 UV–Vis) as described in^29^. The assay medium (1 ml per assay) contained 20 mM Mops/Tris, pH 8.0, 0.2 mM EDTA, 10 mM CH_3_COOK, 1 mM ATP, 3.2 mM Mg(CH_3_COO)_2_, 1 mM phosphoenolpyruvate, at least 0.3 mM NADH, 0.1 mg/ml pyruvate kinase, 0.1 mg/ml lactate dehydrogenase, 5 mM KCN, and 5 μM carbonyl cyanide 4-(trifluoromethoxy)-phenylhydrazone. As noted before^29^, the rate of ATP hydrolysis by membranes increases to a small degree during assays. This is due, at least in part, to dissociation of a small fraction of EcF_1_ and/or ε from the membranes, since the increase is inhibited by added ε (*e.g.*, Fig. 5). To avoid interference of this increase with the changes of ATPase activity induced by venturicidins, unless indicated otherwise, measurement of ATPase activities were started 60 min after addition of membranes to the complete assay medium. This minimizes further increases in ATPase rate during control assays to < 15%. To keep absorbance between 1.5 and 2 units after the 60-min preincubation, up to 0.2 mM additional NADH was added to the assay medium. In figures displaying activity traces, some traces are shifted vertically for visual clarity.

Protein was measured by a modified Lowry procedure^58^.

## Supplementary Information

Supplementary figures.
