# Supplementary material for: Complex effects of macrolide venturicidins on bacterial F-ATPases likely contribute to their action as antibiotic adjuvants
Source: Sci Rep. 2021 Jul 1;11:13631. doi: 10.1038/s41598-021-93098-8 (PMC8249445; doi:10.1038/s41598-021-93098-8)
Supplement: Supplementary file 1 — Supplementary figures. [file 41598_2021_93098_MOESM1_ESM.pdf]

Supplementary Information

Complex effects of macrolide venturicidins on bacterial F-ATPases likely contribute to their action as antibiotic adjuvants

Yakov M. Milgrom and Thomas M. Duncan

Department of Biochemistry & Molecular Biology, SUNY Upstate Medical University, 750 E Adams St, Syracuse, NY 13210, USA

Table of Contents:

Pages S2 – S5, Supplementary Figures S1 – S4

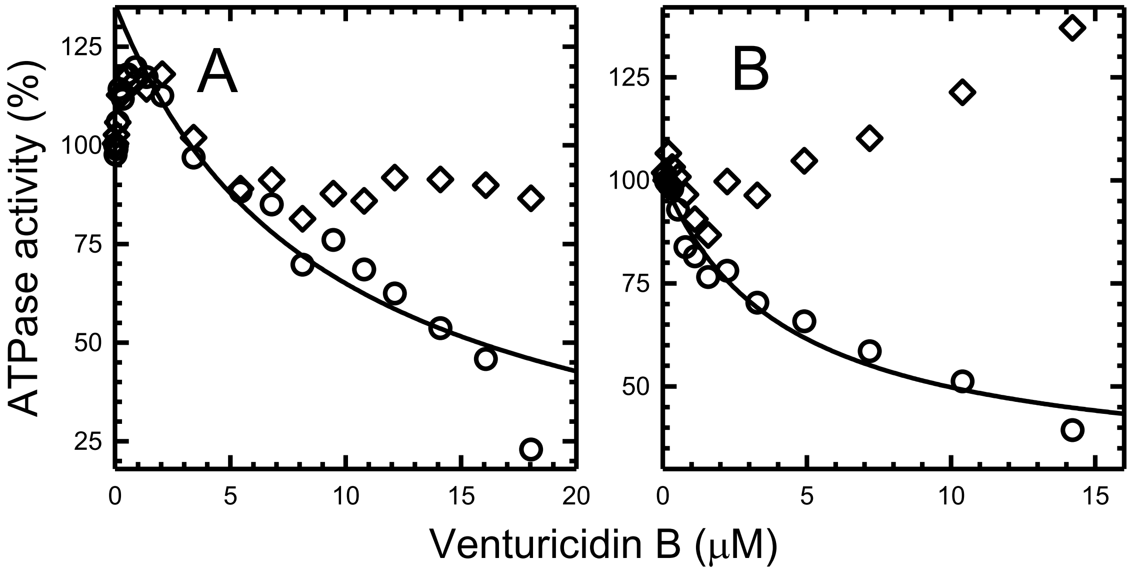

Fig. S1. Concentration-dependence of venturicidin B effects on the rate of ATP hydrolysis by WT *E. coli* membranes. As for Fig. 3, hydrolysis rates were measured early after adding venturicidin B (○) or late (◇) in the absence (**A**) or presence of 38.5 mM selenite (**B**). Assays used 3.36 μg (**A**) or 1.47 μg (**B**) of WT membrane protein. The 100% activity corresponds to 0.82 U/mg (**A**) and 3.36 U/mg (**B**). In each panel, the line shows the best fit of a hyperbolic equation, $y = y_0 + y_1/(1 + x/K_i)$, to the range of early rates (○, *y*) that decrease with increasing ventB concentration (x), which includes points >0.13 μM ventB for panel **A** and all points for panel **B**. Fitting results: panel **A**, y_1 = 136% (±7), K_i = 9 μM (±1), y_0 was fixed as 0; panel **B**, y_1 = 73% (±6), K_i = 4 μM (±1), y_0 = 28% (±7).

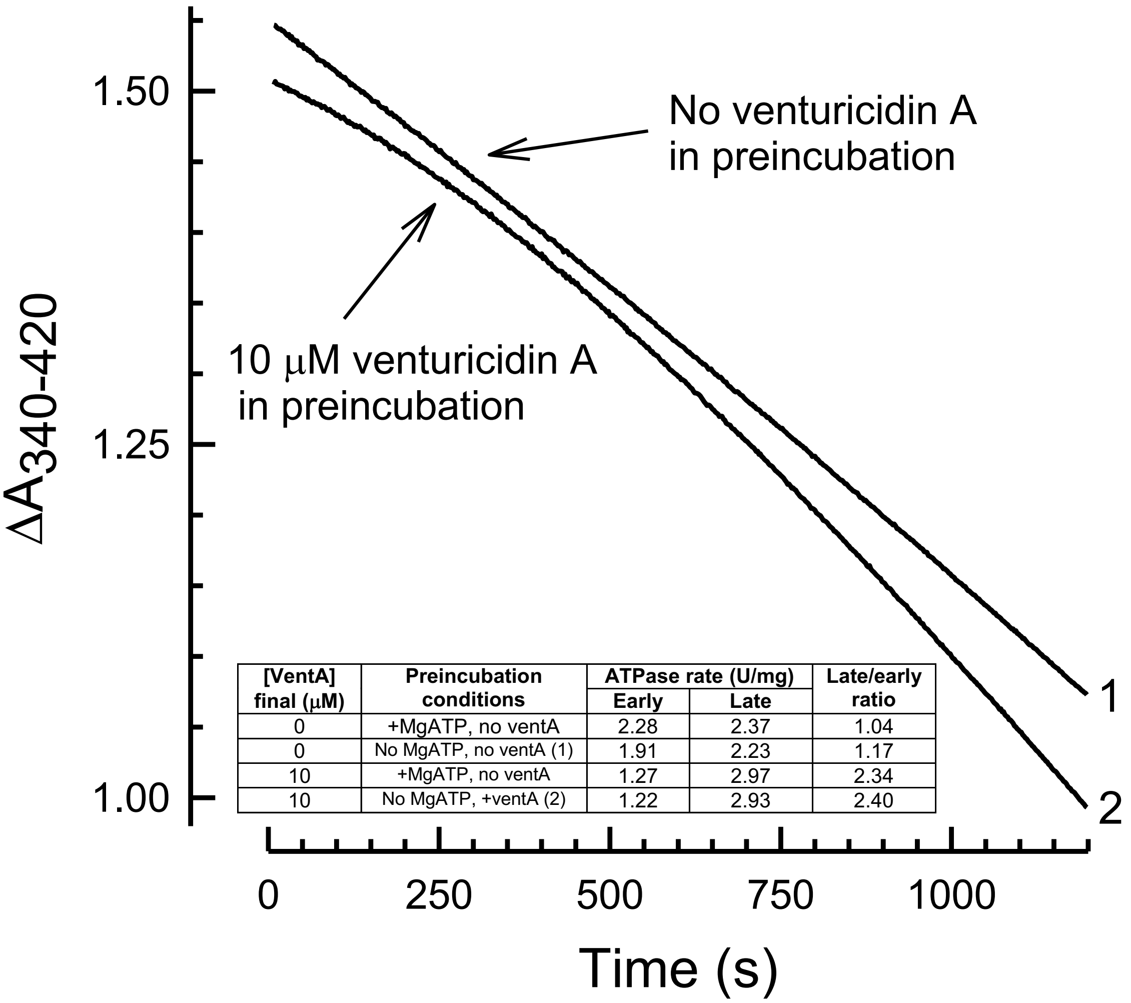

[VentA] final (μM)	Preincubation conditions	ATPase rate (U/mg)		Late/early ratio
		Early	Late	
0	+MgATP, no ventA	2.28	2.37	1.04
0	No MgATP, no ventA (1)	1.91	2.23	1.17
10	+MgATP, no ventA	1.27	2.97	2.34
10	No MgATP, +ventA (2)	1.22	2.93	2.40

Fig. S2. Effects of preincubating *E. coli* membranes with ventA on the kinetics of ATP hydrolysis. WT membranes were incubated at a concentration of 1.78 μg/ml in the ATPase assay medium (see Methods) containing 40 mM selenite and lacking ATP, PEP, and NADH for 30 min at 30°C in the absence (*trace 1*) or presence (*trace 2*) of 10 μM ventA, and each ATPase assay was started by adding 18 μl of a mixture of ATP, PEP, and NADH to 1 ml to obtain final concentrations of 1 mM, 1 mM, and 0.3 mM, respectively. The first 6 s of each assay are omitted for clarity. The inset table compares these early and late rates with those from assays of Fig. 2B that included preincubation of membranes with MgATP (ATPase turnover) but without ventA.

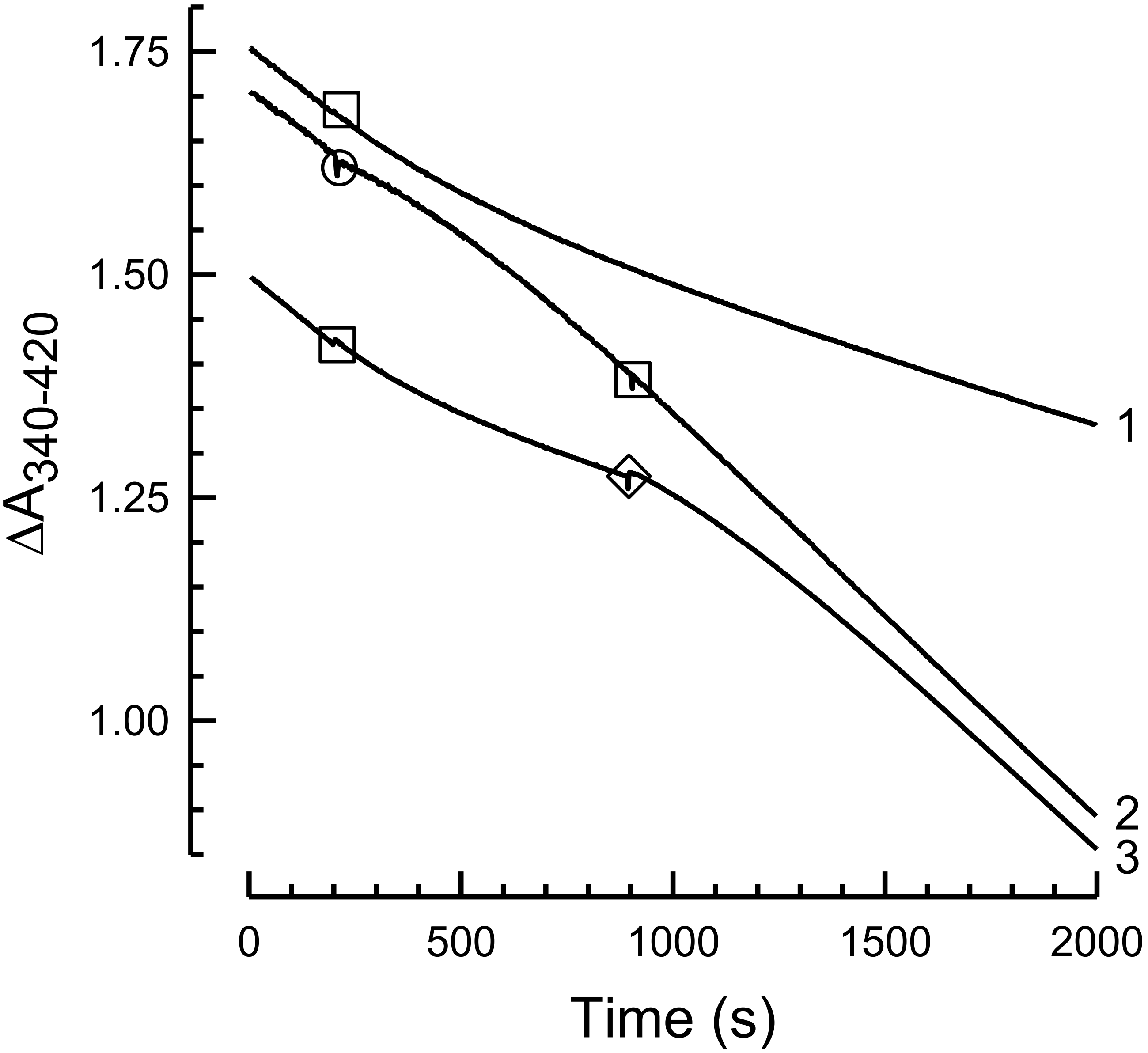

Fig. S3. VentA-induced increase in ATPase activity with selenite present is not blocked by DCCD modification of F_O. ATP hydrolysis was assayed with 38.5 mM selenite present (see Methods), using 1.47 µg of WT *E. coli* membrane protein. DCCD was added to each assay at 0.1 mM (☐) and ventA was added either before DCCD (○, *trace 2*, 10 µM ventA) or after DCCD (◇, *trace 3*, 15 µM ventA). The mean of ATPase activity during the first 3 min of all assays is 2.35 (±0.13) U/mg. During the last 3 min of assays, ATPase activity is 0.85, 2.93, or 2.84 U/mg for *traces 1–3*, respectively. *Traces* are shifted vertically for clarity.

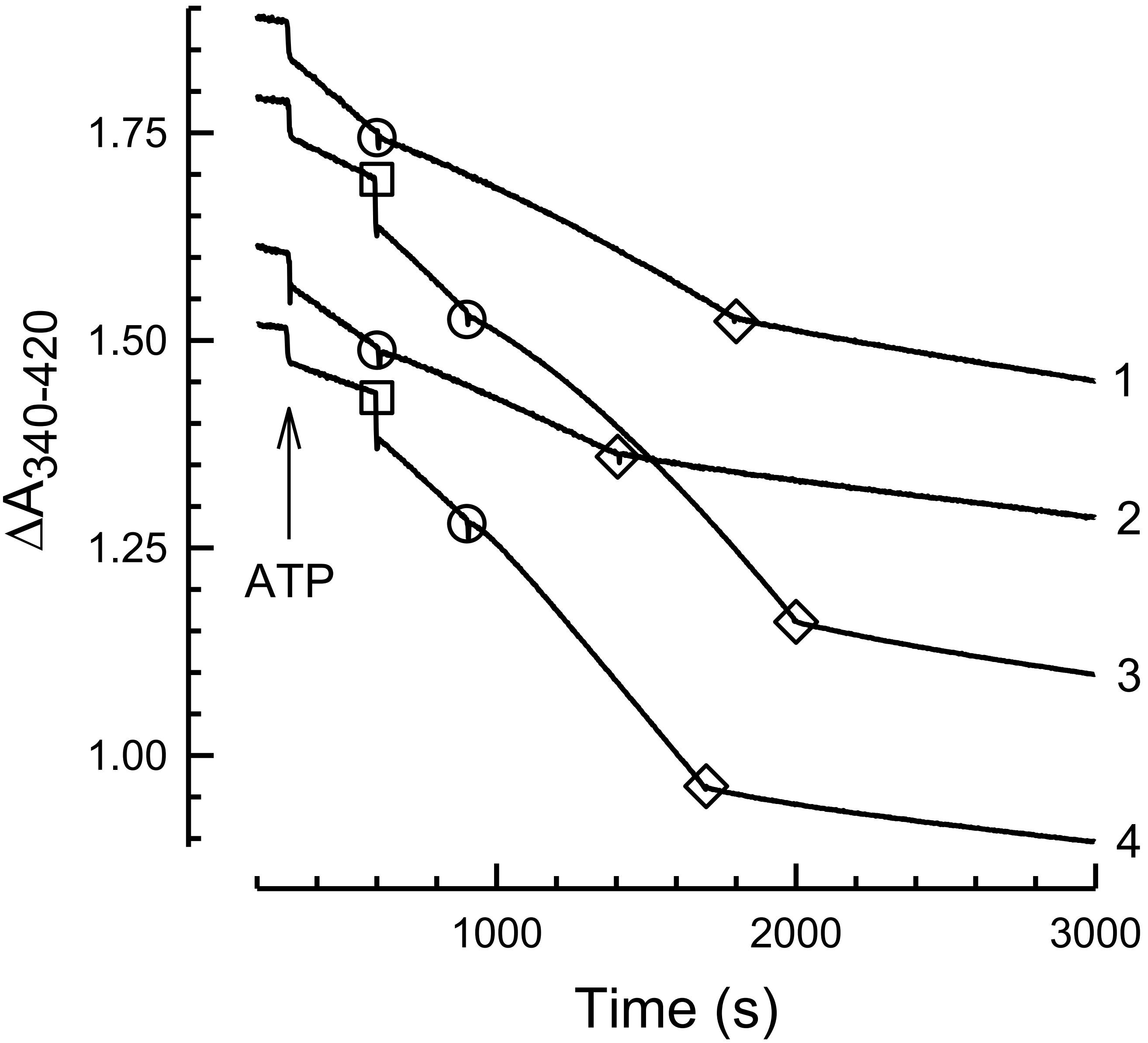

Fig. S4. Addition of WT ε subunit rapidly inhibits ATPase activity that had recovered after exposure of WT or ε88-stop *E. coli* membranes to 10 μM venturicidin A. ATPase assays were started by adding **WT** (*traces 1* and *2*) or **ε88-stop** (*traces 3* and *4*) membranes to the assay medium (see Methods) but lacking ATP. Membrane protein added was 3.15, 1.68, 3.1, and 1.24 μg to start *traces 1–4*, respectively. The arrow indicates addition of ATP (1 mM final) to initiate each assay. Symbols indicate addition of 38.5 mM selenite (□), 10 μM venturicidin A (○), or 88 nM ε (◇). *Traces* are shifted vertically for clarity.
